# *Roseburia intestinalis* Modulates Immune Responses by Inducing M1 Macrophage Polarization

**DOI:** 10.3390/ijms26115049

**Published:** 2025-05-23

**Authors:** Anna Bircher, Egle Katkeviciute, Yasser Morsy, Silvia Lang, Ana Montalban-Arques, Michael Scharl

**Affiliations:** Department of Gastroenterology and Hepatology, University Hospital Zurich, University of Zurich, 8091 Zurich, Switzerland; anna.bircher@usz.ch (A.B.);

**Keywords:** *Roseburia intestinalis*, macrophage polarization, colorectal cancer, microbiota, *Peptostreptococcus stomatis*

## Abstract

In recent years, the gut microbiome has been recognized as one influential factor in cancer development. Particularly in colorectal cancer (CRC), several studies observed a major imbalance of the intestinal microbiota, marked by a reduction in beneficial bacterial species, such as *Roseburia intestinalis*, and an increase in opportunistic pathobionts, like *Peptostreptococcus stomatis*. We previously observed that specific *Eubacteriales*, including *R. intestinalis*, were significantly reduced in CRC patients and have a potent anti-tumor immune effect when applied as oral monotherapy in mice. Here, we investigate the molecular mechanism of *R. intestinalis* on various cell types in vitro, highlighting its potential therapeutic value in CRC. Co-culture experiments with macrophages demonstrated that *R. intestinalis* exposure induced an increase in the M1 phenotype and decreased the M2 phenotype, suggesting macrophage-polarizing properties of these bacteria. *R. intestinalis* also triggered a gene expression profile resembling M1 macrophages and led to distinct chemokine and cytokine secretion in cancer cells, suggesting an immune-activating environment. However, we did not observe direct cytotoxic effects in cancer cells. Our research provides insights into the potential of *R. intestinalis* to activate immune responses, supporting further investigation into its therapeutic role in CRC. These findings underscore the need for deeper studies on the bacterium’s impact on CRC pathogenesis and treatment.

## 1. Introduction

Colorectal carcinoma (CRC) is one of the most common cancers worldwide, with over 1.9 million newly diagnosed cases and 935,000 estimated deaths in 2020. Thus, CRC represented 10% of global cancer incidence and 9.4% of all cancer-related deaths in 2020 [1,2,3]. Incidence and mortality rates vary geographically, with the highest rates observed in more industrialized regions such as Europe, Australia/New Zealand and North America [2]. Since the prevalence of CRC is continuously growing dramatically, the incidence of CRC worldwide is predicted to increase up to 3.2 million new cases and 1.6 million deaths per year in 2040 (corresponding to an increase of 73%) [3].

The human gastrointestinal (GI) microbiota is an intricate ecosystem, consisting of a diverse community of microorganisms including bacteria, fungi, archaea, protozoa, bacteriophages and viruses, residing on a total mucosal area of 200–300 m^2^ [4]. It has been estimated that a human GI tract harbors 10^14^ microorganisms, which corresponds to ten times the number of human cells and represents more than 100 times the amount of genomic material compared to the material of human origin [5]. In total, over 3000 bacterial species have been identified in human feces, primarily consisting of anaerobic bacteria [6]. Individuals typically harbor around 200 different species in their GI tract, with a considerable variability in the abundance of these species among different people [7]. Only about 30% of the bacterial population is shared across the population, forming what is referred to as the common core microbiota [8]. The GI microbiota benefits the host through various physiological functions, including strengthening gut integrity, shaping the intestinal epithelium, fermenting non-digestible carbohydrates, absorbing nutrients, biosynthesizing essential vitamins, protecting against pathogens and regulating the host’s immune system [4,9]. Hence, a disturbance in the microbial composition, known as dysbiosis, can potentially disrupt these beneficial mechanisms [10].

In recent years, the gut microbiome has been recognized as one influential factor in cancer development. In fact, cancer patients harbor an altered microbiome that might contribute to cancer pathogenesis, as well as to treatment resistance [11,12]. Particularly, in the context of CRC pathogenesis, several studies showed a major structural imbalance of the intestinal microbiota towards a reduction in butyrate-producing bacteria and an increase in opportunistic pathogens, such as *P. stomatis* [13,14,15,16]. In contrast, there are members of the native microbiota that have been attributed with beneficial effects for the host. Particularly, representatives of the *Lactobacillus* or *Bifidobacterium* genera and the butyrate-producing community are associated with anti-inflammatory and anti-tumorigenic properties [11]. In fact, recent studies have shown that specific members of the gut microbiota are able to increase the efficacy of immune checkpoint inhibitor (ICI) immunotherapy in different types of cancers, such as melanoma, sarcoma or CRC [17,18,19,20,21]. In a previous study, we found that specific *Clostridiales* (recently reclassified to *Eubacteriales*), which are significantly reduced in patients with CRC, induce a potent anti-tumor immune response when applied as an oral monotherapy to mice bearing tumors [16]. While these studies clearly show the link between the intestinal microbiome and the efficacy of cancer immunotherapy, the purpose of this work is to further investigate the molecular mechanisms behind this relation, which remain largely unknown.

*R. intestinalis* is a commensal microorganism that ranks among the top 20 most prevalent bacteria, constituting 0.9–5.0% of the total microbiota [8,22]. Numerous clinical investigations involving human cohorts and established disease models have extensively documented the protective attributes of *Roseburia* spp., particularly of *R. intestinalis*. Many publications suggest that a decreased presence of this bacterium in the feces is associated with various diseases, including inflammatory bowel disease [23,24], type 2 diabetes mellitus [25], antiphospholipid syndrome [26], atherosclerosis [27,28] and CRC [16]. Importantly, *R. intestinalis* has displayed beneficial effects through different molecular mechanisms involving its main metabolite butyrate, other extracellular components, flagellin or other bacterial structural components [24,29,30]. Of note, we have recently demonstrated that *R. intestinalis* induces a strong anti-tumor immune response and, if orally applied, is a highly effective anti-cancer therapy in several mouse models [16]. The research problem lies in the fact that *R. intestinalis* is an obligate anaerobe, making it difficult to cultivate. Therefore, the main hurdle in conducting thorough investigations primarily lies in the demanding culture conditions of *R. intestinalis* strains, in addition to the lack of well-designed clinical trials to conclusively demonstrate its protective role and therapeutic potential [31].

Therefore, the aim of this study was to identify the specific molecular mechanism of action that links the oral supplementation of *R. intestinalis* bacteria to the observed anti-tumor immune response and further tumor shrinkage. We hypothesize that *R. intestinalis* triggers a strong immune response by potentially polarizing macrophages toward an M1 phenotype.

## 2. Results

### 2.1. R. intestinalis Treatments Induce M1 Polarization of Macrophages

**Figure 1:**
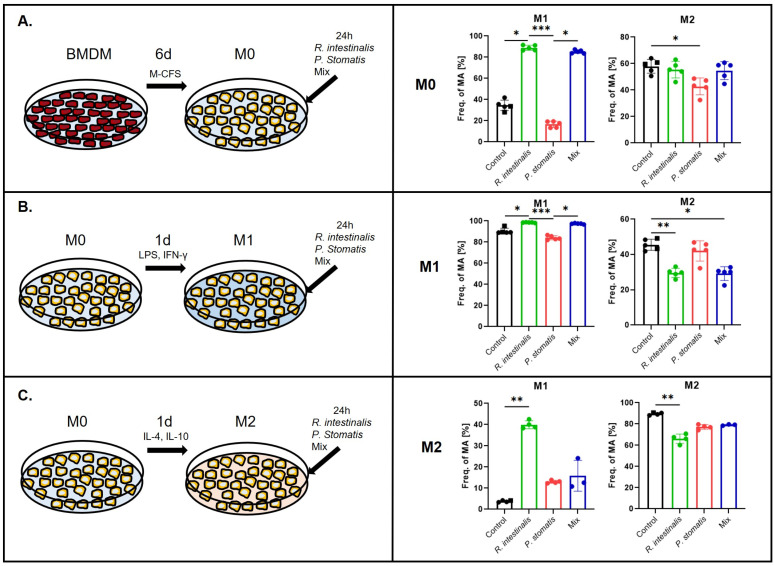
Polarization of macrophages following bacterial stimulation. Experimental setup and flow cytometry analysis of (**A**) M0, (**B**) M1 and (**C**) M2 pre-differentiated macrophages and YCFA medium (control), *R. intestinalis*, *P. stomatis* or bacterial mix stimulation with M1 polarization indicated by CD80+, CD86+ double-positive macrophages and M2 double-positive for CD206+, CD163+. Frequency of macrophages (Freq. of MA (F4/80+ CD64+)) as the percentage of cells in this population out of the F4/80 and CD64 double-positive macrophage parent population. Five different experiments were performed, consisting of *n* = 5 in all groups. The data presented are from one representative subset. *p*-value was determined with Kruskal–Wallis and Dunn’s correction for multiple comparison with * *p* < 0.05, ** *p* < 0.01 and *** *p*< 0.001. With BMDM=Bone marrow-derived recruited macrophages, M0 = undifferentiated macrophages, M1 = pro-inflammatory macrophages and M2 = anti-inflammatory macrophages.

To assess the impact of *R. intestinalis* and *P. stomatis* on immune cells, aerobic co-culture experiments were conducted. However, this approach compromised the viability of the anaerobic bacterial culture. We used *P. stomatis* as a control, since we had previously shown that oral administration of this species induces tumor development in vivo [16]. BMDMs, either in their undifferentiated state (M0) or induced into M1 or M2 phenotypes, were subjected to co-culture with *R. intestinalis*, *P. stomatis* or a combination of both bacteria (mix) for a duration of 24 h. Flow cytometry analysis revealed that co-culturing M0, M1 and M2 macrophages with *R. intestinalis* led to a significant increase in the M1 phenotype, as indicated by higher levels of the CD80+ and CD86+ markers (Figure 1, Supplementary Figure S1). This increase was statistically significant when compared to both the control group and the group treated with *P. stomatis*. A similar trend was observed with the mix treatment in the cases of M0 and M1, demonstrating that the effect of *R. intestinalis* was still visible after combination with *P. stomatis*. Moreover, the analysis revealed a significant reduction in the M2 phenotype, as characterized by the CD206+ and CD163+ markers. This reduction was observed in M1 and M2 macrophages stimulated with *R. intestinalis,* M1 macrophages stimulated with the mix treatment and M0 macrophages stimulated with *P. stomatis* (Figure 1). These data indicate that *R. intestinalis* promotes the plasticity and differentiation of pro-inflammatory M1 macrophages and reduces the amount of differentiation of anti-inflammatory M2 macrophages.

**Figure 2:**
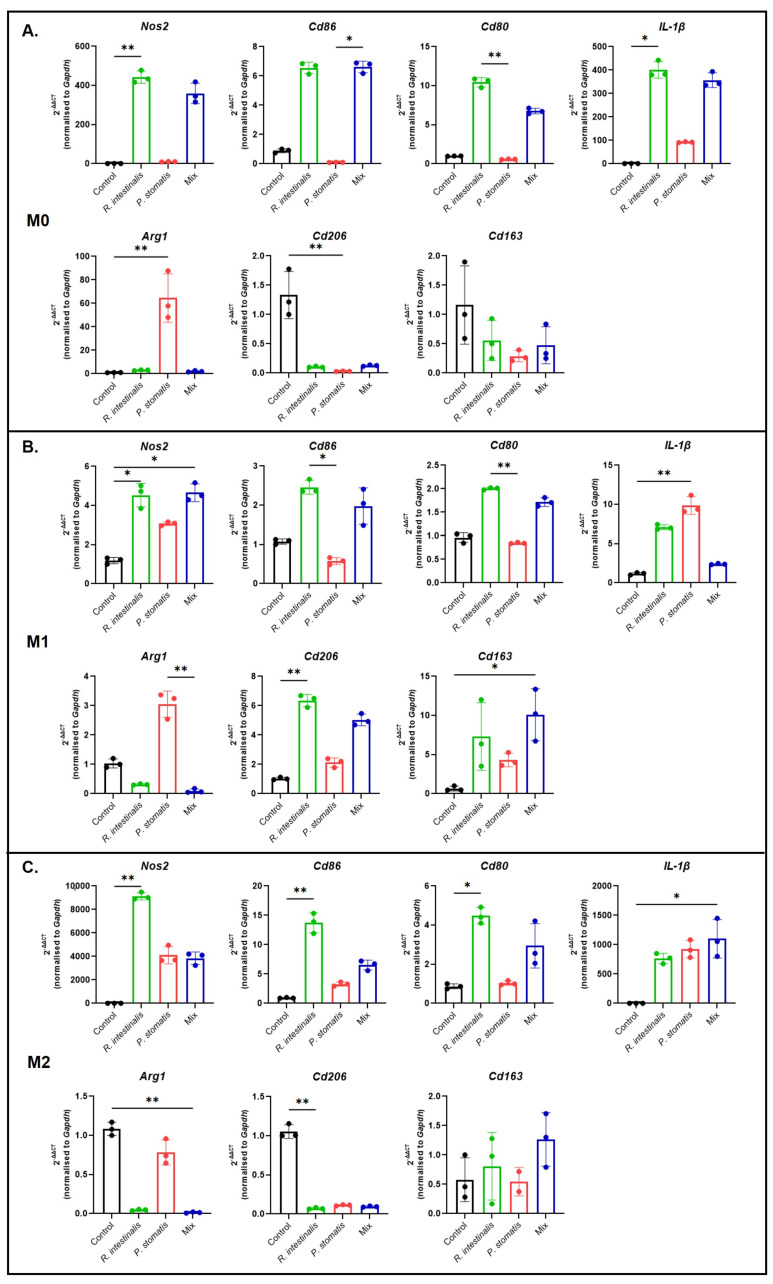
Polarization of macrophages following bacterial stimulation. qPCR results of *Nos2*, *Cd86*, *Cd80*, *IL-1b*, *Arg1*, *Cd206* and *Cd163* expression from (**A**) M0, (**B**) M1 and (**C**) M2 pre-differentiated macrophages and YCFA medium (control), *R. intestinalis*, *P. stomatis* or bacterial mix stimulation. Five different experiments were performed, consisting of *n* = 3 in all groups. The data presented are from one representative subset. *p*-value was determined with Kruskal–Wallis and Dunn’s correction for multiple comparison with * *p* < 0.05 and ** *p* < 0.01. With M0 = undifferentiated macrophages, M1 = pro-inflammatory macrophages and M2 = anti-inflammatory macrophages.

To support our flow cytometry data, we next performed qPCR analysis. Here, the data fully corroborated our findings, revealing consistent outcomes (Figure 2). Specifically, and most prominently, treatment with *R. intestinalis* increased the mRNA expression levels of the M1 markers *Nos2*, *Cd86* and *Cd80*, as well as of the pro-inflammatory cytokine *IL-1b*, in all three macrophage subtypes. A similar pattern emerged with the mix stimulation, again suggesting that the *R. intestinalis*-mediated effect is predominant when combining those two bacteria. With respect to the M2 markers, *Arg1*, *Cd206* and *Cd163*, *R. intestinalis* treatment reduced their levels in M0 and M2 but not consistently in M1 macrophages. In the latter, *Arg1* levels were reduced, while *Cd206* and *Cd163* levels were enhanced. These effects were also visible with the mix.

Conversely, stimulation with *P. stomatis* consistently reduced the levels of the M1 markers across all three macrophage subtypes and increased the pro-inflammatory cytokine *IL-1b* in M1 and M2. With respect to the *P. stomatis*-induced levels of the M2 marker *Arg1* across all macrophage conditions, it had only limited effects on *Cd163* and *Cd206* (Figure 2).

### 2.2. R. intestinalis Induces a Characteristic Gene Expression Pattern in Macrophages

**Figure 3:**
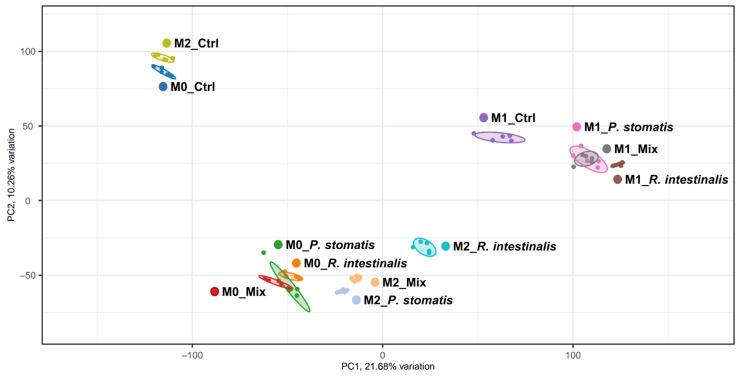
PCoA plot of macrophages stimulated with bacteria. RNA sequencing was conducted in M0, M1 and M2 pre-differentiated macrophages with either YCFA medium (control), *R. intestinalis*, *P. stomatis* or bacterial mix stimulation. Five different experiments were performed, consisting of *n* = 5 in all groups. The data presented are from one representative subset. With M0 = undifferentiated macrophages, M1 = pro-inflammatory macrophages and M2 = anti-inflammatory macrophages.

To explore the impact of *R. intestinalis* and *P. stomatis* on the polarization of macrophages at the RNA level, we performed bulk RNA sequencing of M0, M1 and M2 pre-differentiated macrophages stimulated for 24 h with *R. intestinalis*, *P. stomatis* or a combination thereof. In a Principal Coordinate Analysis (PCoA), M2 macrophages treated with *R. intestinalis* exhibited a slight clustering towards the M1 control macrophages, suggesting an increased degree of similarity between these two groups (Figure 3). Additionally, the M0 control group clustered together with the M2 control group.

**Figure 4:**
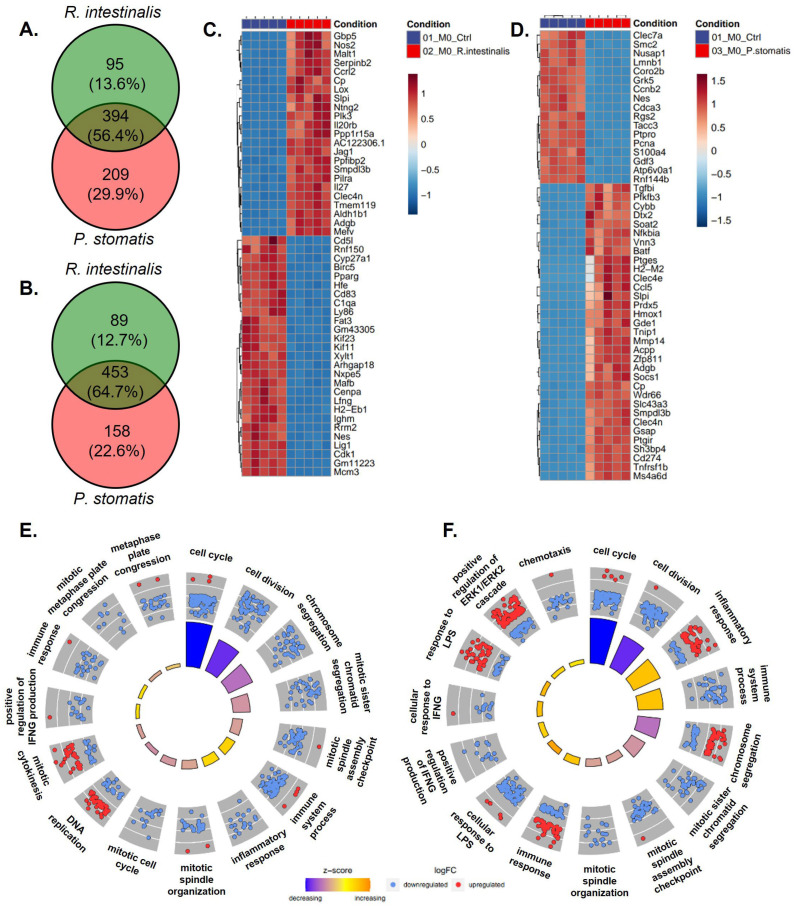
RNA sequencing with Venn diagram, heatmap and Gene Ontology (GO) enrichment analysis of M0 differentiated bacterial-treated macrophages. Venn diagram of differentially expressed genes of M0 macrophages stimulated with *R. intestinalis* or *P. stomatis*, compared to control group with (**A**) upregulated and (**B**) downregulated genes. Heatmaps showing the top differentially expressed genes (DEGs) of M0 macrophages stimulated with (**C**) *R. intestinalis* or (**D**) *P. stomatis*, compared to control group. DAVID visualization of enriched GO terms of the biological process from the DEGs in M0 differentiated macrophages treated with (**E**) *R. intestinalis* or (**F**) *P. stomatis*, compared to control group. With M0 = undifferentiated macrophages.

**Figure 5:**
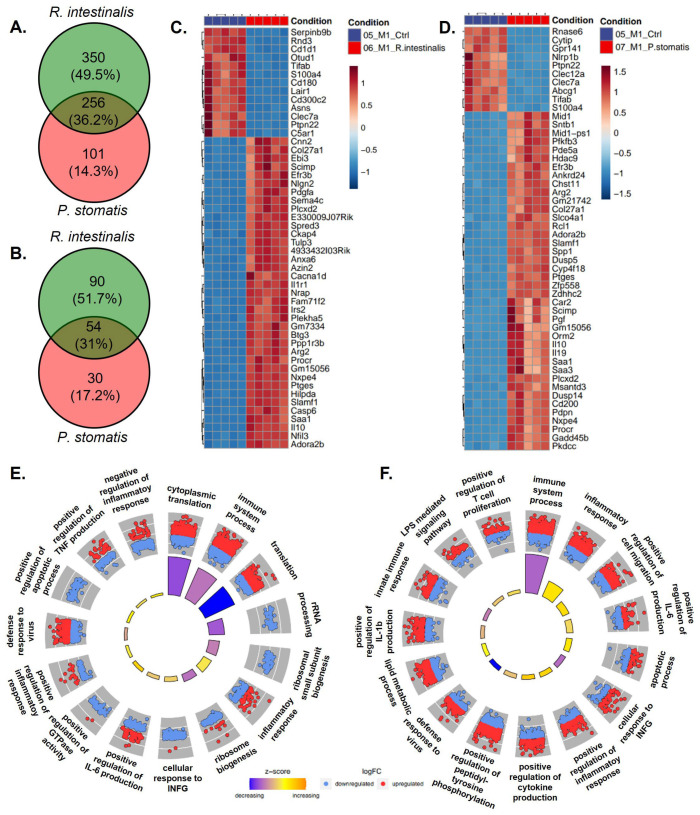
RNA sequencing with Venn diagram, heatmap and Gene Ontology (GO) enrichment analysis of M1 differentiated bacterial-treated macrophages. Venn diagram of differentially expressed genes of M0 macrophages stimulated with *R. intestinalis* or *P. stomatis*, compared to control group with (**A**) upregulated and (**B**) downregulated genes. Heatmaps showing the top differentially expressed genes (DEGs) of M1 macrophages stimulated with (**C**) *R. intestinalis* or (**D**) *P. stomatis*, compared to control group. DAVID visualization of enriched GO terms of the biological process from the DEGs in M1 differentiated macrophages treated with (**E**) *R. intestinalis* or (**F**) *P. stomatis,* compared to control group. With M1= pro-inflammatory macrophages.

**Figure 6:**
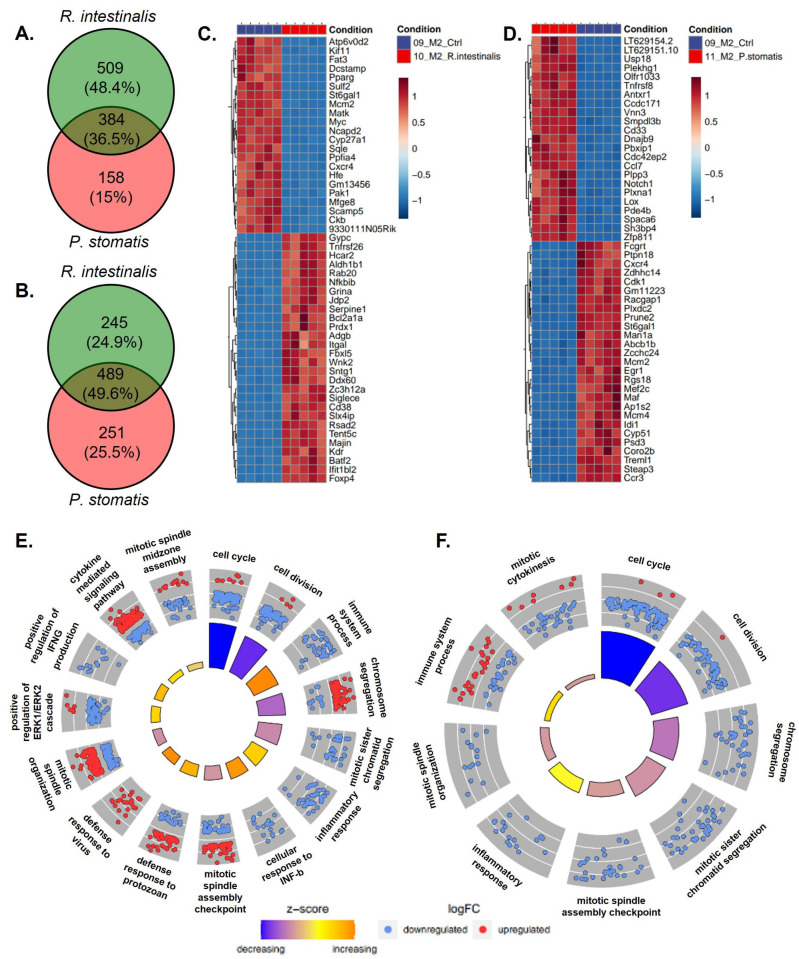
RNA sequencing with Venn diagram, heatmap and Gene Ontology (GO) enrichment analysis of M2 differentiated bacterial-treated macrophages. Venn diagram of differentially expressed genes of M2 macrophages stimulated with *R. intestinalis* or *P. stomatis*, compared to control group with (**A**) upregulated and (**B**) downregulated genes. Heatmaps showing the top differentially expressed genes (DEGs) of M2 macrophages stimulated with (**C**) *R. intestinalis* or (**D**) *P. stomatis*, compared to control group. DAVID visualization of enriched GO terms of the biological process from the DEGs in M2 differentiated macrophages treated with (**E**) *R. intestinalis* or (**F**) *P. stomatis*, compared to control group. With M2 = anti-inflammatory macrophages.

The Venn diagram analysis of differentially expressed genes (DEGs) indicated a bacteria-specific gene expression profile associated with either *R. intestinalis* or *P. stomatis* treatment when compared to the control group. Moreover, there were differences in gene expression between *R. intestinalis* and *P. stomatis* treatments, with 13.6% of genes being differentially expressed in an *R. intestinalis*-specific manner in M0 upregulated, 12.7% in M0 downregulated (Figure 4A,B), 49.5% in M1 upregulated, 51.7% in M1 downregulated (Figure 5A,B), 48.4% in M2 upregulated and 24.9% in M2 downregulated (Figure 6A,B). The heatmap from the *R. intestinalis-*(Figure 4C, Figure 5C and Figure 6C; Supplementary Figure S2A–C) and *P. stomatis* (Figure 4D, Figure 5D and Figure 6D; Supplementary Figure S2A–C)-treated macrophages revealed a unique gene pattern in comparison to the control group, indicating specific gene changes triggered by each bacteria. A DAVID visualization of top significant Gene Ontology (GO) terms for biological process analysis showed a bacteria-specific gene expression profile for both *R. intestinalis* (Figure 4E, Figure 5E and Figure 6E) and *P. stomatis* (Figure 4F, Figure 5F and Figure 6F) within all M0, M1 and M2 macrophage populations. Of note, based on the pathway analysis (Figure 4E,F, Figure 5E,F and Figure 6E,F), none of the significantly upregulated or downregulated pathways in our bacteria groups were associated with phagocytosis, thus suggesting that phagocytosis would be activated by our two bacteria in the macrophages.

In summary, on multiple analytic levels, our co-culture experiments involving macrophages and bacteria revealed that exposure to *R. intestinalis* induced a significant increase in the M1 phenotype and a concurrent decrease in the M2 phenotype within macrophage populations. A similar trend was observed with the mix treatment, albeit with a lower increase in M1. Further, *R. intestinalis* induced a specific gene expression profile within macrophage populations. A close clustering was observed between *R. intestinalis*-stimulated M2 macrophages and M1 control samples. These findings underscore the potential influence of *R. intestinalis* on macrophage polarization, repolarization and immune responses.

### 2.3. R. intestinalis Enhances M1 Macrophage Polarization After Co-Culture with MC-38 CRC Cells

To explore the influence of *R. intestinalis* and *P. stomatis* on immune cells in a cancerous context, we next conducted co-culture experiments involving MC-38 cells and BMDMs in their undifferentiated M0 state. The cells were co-cultured with *R. intestinalis*, *P. stomatis* or a combination thereof for a 24 h period (MC-38 + M0).

**Figure 7:**
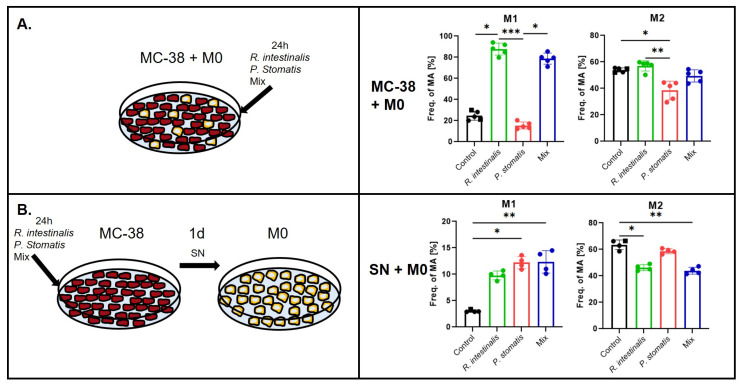
Polarization of macrophages in an in vitro cancer setting following bacterial stimulation. Experimental setup and flow cytometry analysis with M1 polarization indicated by CD80 and CD86 double-positive macrophages and M2 double-positive for CD206 and CD163 of (**A**) MC-38 and M0 co-cultures and YCFA medium (control), *R. intestinalis*, *P. stomatis* or bacterial mix stimulation (MC-38 + M0) or (**B**) supernatant collected from MC-38 cells stimulated with YCFA medium (control), *R. intestinalis*, *P. stomatis* or bacterial mix and M0 co-cultures (SN + M0). Frequency of macrophages (Freq. of MA) as the percentage of cells in this population out of the F4/80 and CD64 double-positive macrophage parent population. Three different experiments were performed, consisting of *n* = 5 in all groups. The data presented are from one representative subset. *p*-value was determined with Kruskal–Wallis and Dunn’s correction for multiple comparison with * *p* < 0.05, ** *p* < 0.01 and *** *p*< 0.001 With M0 = undifferentiated macrophages, M1 = pro-inflammatory macrophages, M2 = anti-inflammatory macrophages, MC-38 cells = C57BL6 murine colon adenocarcinoma cells and SN = cell culture supernatant.

**Figure 8:**
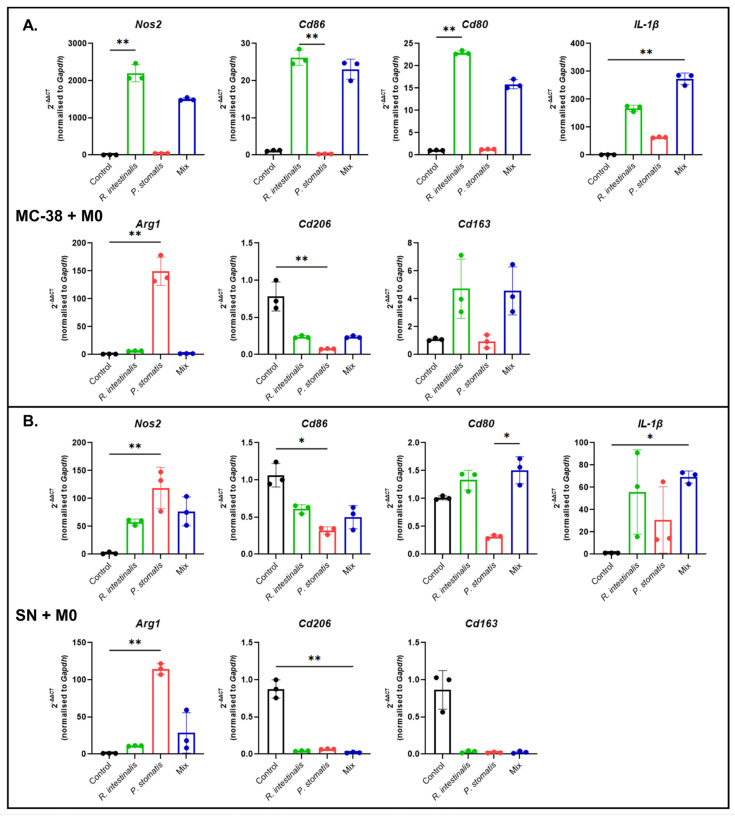
Polarization of macrophages in an in vitro cancer setting following bacterial stimulation. qPCR results of *Nos2*, *Cd86*, *Cd80*, *IL-1b*, *Arg1*, *Cd206* and *Cd163* expression from (**A**) MC-38 and M0 co-cultures and YCFA medium (control), *R. intestinalis*, *P. stomatis* or bacterial mix stimulation (MC-38 + M0) or (**B**) supernatant collected from MC-38 cells stimulated with YCFA medium (control), *R. intestinalis*, *P. stomatis* or bacterial mix and M0 co-cultures (SN + M0). Three different experiments were performed, consisting of *n* = 3 in all groups. The data presented are from one representative subset. *p*-value was determined with Kruskal–Wallis and Dunn’s correction for multiple comparison with * *p* < 0.05 and ** *p* < 0.01. With M0 = undifferentiated macrophages, M1 = pro-inflammatory macrophages, M2 = anti-inflammatory macrophages, MC-38 cells = C57BL6 murine colon adenocarcinoma cells and SN = cell culture supernatant.

Flow cytometry analysis unveiled compelling results: the MC-38 + M0 co-culture experiments demonstrated a significant increase in M1 macrophage differentiation upon *R. intestinalis* stimulation, surpassing the effects of *P. stomatis* stimulation and the control groups (Figure 7A, Supplementary Figure S1). Additionally, an increase in M1 macrophages was observed with the mix stimulation. Conversely, *P. stomatis* co-culture led to a decrease in M2 macrophages compared to all other groups. qPCR analysis in the MC-38 + M0 experiments revealed increased expressions of *Nos2*, *Cd86*, *Cd80*, *IL-1b* and *Cd163*, alongside a decrease in *Cd206* expression, following *R. intestinalis* stimulation (Figure 8A). The mix stimulation resulted in increased *Nos2*, *Cd86*, *Cd80*, *IL-1b* and *Cd163* expression, along with decreased *Cd206* expression. Furthermore, there was an increase in *Arg1* expression and a decrease in *Cd206* expression with *P. stomatis* stimulation.

Additionally, further co-culture experiments were performed to investigate the impact of bacteria-stimulated MC-38 cells and the subsequent release of chemokines and cytokines on macrophages. These experiments involved a co-culture of bacteria and MC-38 cells for 24 h, followed by a stimulation of M0 macrophages with the supernatant (SN) of the MC-38 cell culture medium for another 24 h (SN + M0). Here, co-culturing the bacterial supernatant with M0 macrophages led to increased M1 differentiation and a significant decrease in M2 macrophages within the *R. intestinalis* group (Figure 7B). Similar effects were observed with the mix stimulation, which showed an increase in M1 and a decrease in M2 macrophages. An increase in M1 macrophages was noted in the *P. stomatis* group.

In the SN + M0 experiments, stimulation with *R. intestinalis* led to increased *Nos2*, *Cd80* and *IL-1b* expression, accompanied by decreased *Cd206* and *Cd163* expression (Figure 8B). Similarly, the mix stimulation led to increased *Nos2*, *Cd80* and *IL-1b* expression and decreased *Cd206* and *Cd163* expression. Stimulation with *P. stomatis* resulted in increased *Nos2* and *Arg1* expression while decreasing *Cd86, Cd80*, *Cd206* and *Cd163* expression. These experiments show a polarization towards M1 with *R. intestinalis* treatment in a cancer setting but a bacteria-unspecific priming in the experiments involving MC-38 and bacterial supernatant.

### 2.4. R. intestinalis Impact on MC-38 Cell Chemokine and Cytokine Production

**Figure 9:**
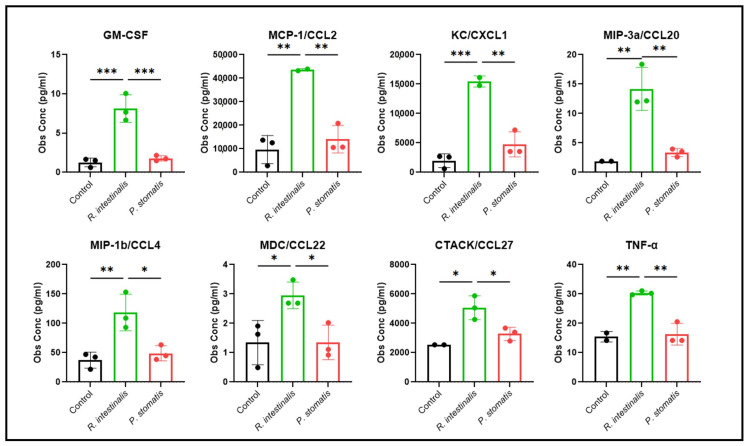
Bio-Plex multiplex immunoassay of MC-38 cells. Chemokine and cytokine expression levels of supernatant from MC-38 cells co-cultured with *R. intestinalis* or *P. stomatis*, measured with a Bio-Plex multiplex immunoassay. Two different experiments were performed, consisting of *n* = 3 in all groups. The data presented are from one representative subset. *p*-value was determined with Kruskal–Wallis and Dunn’s correction for multiple comparison with * *p* < 0.05, ** *p* < 0.01 and *** *p* < 0.001. With Obs Conc = observed concentration.

To assess the impact of *R. intestinalis* and *P. stomatis* on the secretion of chemokines and cytokines from MC-38 and M0 cells, we incubated either MC-38 cells, M0 or a co-culture of MC-38 and M0 cells with either *R. intestinalis* or *P. stomatis* over a 24 h period. Using a Bio-Plex multiplex immunoassay approach, the supernatant of the cell cultures was examined to discern the chemokine and cytokine release profiles (Figure 9 and Table 1). Notably, *R. intestinalis* treatment led to a distinctive pattern of chemokine and cytokine secretion from MC-38 cells. Specifically, the levels of the chemokines/chemoattractant proteins CCL2, CXCL1, CCL20, CCL4, CCL22 and CCL27, as well as of GM-CSF and TNF-α, were increased compared to the control conditions and the *P. stomatis* treatment in the supernatant from MC 38 cells. However, neither M0 cells nor the co-culture of MC-38 and M0 cells showed noticeable chemokine and cytokine releases that could be attributed to a specific bacterial treatment (Table 1).

## 3. Discussion

In our research work, we demonstrated that *R. intestinalis* fosters the differentiation of macrophages into pro-inflammatory M1 macrophages that are important for anti-tumor immunity. This well matches our previous in vivo findings and those of others, demonstrating that the oral supplementation of *R. intestinalis* exerts a strong anti-tumor effect in several mouse models [16,23,27,32]. This is of particular importance since numerous investigations involving *R. intestinalis* demonstrated primarily correlations and described a consistent reduction in the abundance of *R. intestinalis* in patients suffering from diseases such as amyotrophic lateral sclerosis, pancreatic ductal adenocarcinoma, ulcerative colitis and CRC [32,33,34,35,36,37,38]. However, the precise role of *R. intestinalis* and its metabolites, or the broader implications of *R. intestinalis* in fostering gut microenvironment recovery and improving disease pathophysiology, remain unclear. Several factors contribute to this lingering uncertainty, especially the intricacies of quantifying microbiota composition and the challenges associated with anaerobic bacteria cultivation. To address these issues, we investigated a more isolated and direct impact of *R. intestinalis* on immune, cancer and/or epithelial cancer cells in an in vitro approach.

Mammalian cells inherently require oxygen for survival, while *R. intestinalis* bacteria thrive under anaerobic conditions but have a limited viability of no more than one hour when exposed to oxygen. Consequently, our initial task involved ascertaining the survival time of mammalian cells under anaerobic conditions. Our assessments revealed that MC-38 cells and BMDMs remained viable for up to six hours in an anaerobic environment (Supplementary Figure S3). Our attempt to perform experiments with a duration of up to six hours with bacterial stimulation in the absence of oxygen did not yield discernible differences via flow cytometry analysis (Author’s unpublished data [39]). These results are in line with the notion that a timeframe of 6 h suffices to induce signaling changes and various pathway activations but is insufficient to produce discernible effects at the protein level [40]. Additionally, while mammalian cells remain alive, they could be exposed to considerable stress caused by the anaerobic condition and, therefore, probably do not represent a healthy physiological state. Therefore, all subsequent cell culture experiments were conducted under aerobic conditions, recognizing that bacterial cells would no longer be viable.

As a consequence, we decided to narrow our objective and started the in vitro experiments with a focus on macrophages. The GI tract stands as the largest mucosal surface in the body and constitutes the single biggest compartment of the immune system. It is constantly exposed to an array of foreign antigens and is required to discriminate between harmful and innocuous antigens to mount suitable responses. While robust protective immune responses are essential against pathogenic insults, similar responses against dietary proteins or commensal bacteria can provoke autoimmunity and chronic inflammatory disorders [41]. Macrophages are distributed throughout the GI tract mucosa, primarily within the lamina propria, which is adjacent to the epithelial monolayer [42]. *R. intestinalis* predominantly colonizes the cecal and colonic mucus. It relies on its flagellum to traverse the colonic mucus layer, which allows interactions with macrophages and epithelial cells [43,44]. Consequently, it is essential to investigate the interplay between macrophages and *R. intestinalis.*

Macrophages exhibit various activation states influenced by factors such as microbial invasions, tissue injury and polarized adaptive T-cell responses [45]. The two main activation phenotypes are M1 (pro-inflammatory) and M2 (anti-inflammatory). M1 is marked by the expression of pro-inflammatory cytokines, inducible nitric oxide synthase (iNOS or NOS2), generation of reactive nitrogen intermediates (RNI) and reactive oxygen species (ROS), promotion of Th1 responses and potent microbicidal and tumoricidal activity. M2 macrophages, on the other hand, contribute to tissue remodeling, dampening of inflammation, parasite clearance and immunoregulation [46]. However, macrophage activation is more complex than can be explained by these two categories. Recent findings indicate their adaptability and ability to transition between inflammatory and resolution phases. In essence, M1 and M2 macrophage polarizations represent extremes within a spectrum of activation states, illustrating the dynamic nature of macrophage responses [47]. Over the past twenty years, mounting evidence has demonstrated that the tumor microenvironment (TME) exerts a critical influence on tumor development [48]. Tumor-associated macrophages (TAMs) represent a significant portion of immune cells in the TME and are especially prevalent in tumors such as CRC [49,50]. Research has demonstrated that TAMs are linked to poor prognosis in the majority of solid tumors [51,52]; however, their role is somewhat more complicated in CRC, where reprogramming TAM polarization could enhance tumor immunotherapy [48]. TAMs primarily adopt an M2-like macrophage phenotype within the TME. They bolster tumor immunosuppression through mechanisms such as driving tumor angiogenesis and indirectly modulating immune cell interactions [53]. The dynamic nature of macrophage polarization suggests a promising avenue for their utilization as therapeutic targets. This proposition gains particular significance within the colon, where a multitude of microbes and their products can significantly influence macrophage behavior.

Our co-culture experiments demonstrated that exposure to *R. intestinalis* led to a significant increase in the M1 macrophage phenotype and a simultaneous reduction in the M2 phenotype within macrophage populations. While the mixed treatment exhibited a similar trend, it was less pronounced in terms of M1 enhancement, probably due to the counteracting effect of *P. stomatis*. These findings underscore the potential influence of *R. intestinalis* on macrophage polarization and immune responses. It is important to note that the M1 and M2 dichotomy represents a continuum, and as such, it is not always straightforward to discern a clear-cut pattern [54], potentially explaining the less pronounced polarization observed with the mix treatment. Macrophages possess the capacity to act as a defense line against microbial invasions and tumor cell recognition, with M1 macrophages demonstrating pro-inflammatory and anti-cancer properties [54]. Given the commensal nature of *R. intestinalis* and its constant exposure to colonic macrophages, it is more plausible that these macrophages are activated to combat cancer. Moreover, there is a paucity of studies demonstrating a negative impact of *R. intestinalis*; the existing literature primarily emphasizes its protective role rather than adverse health effects [31]. Thus, it seems improbable that *R. intestinalis*-stimulated macrophages would induce inflammation and eliminate external bacteria.

RNA sequencing analysis of macrophages and bacteria co-culture experiments further substantiated our findings, revealing a distinct gene expression profile induced by *R. intestinalis* in macrophage populations. The PCoA demonstrated a slight resemblance between *R. intestinalis*-stimulated M2 macrophages and M1 control samples. This alignment with qPCR and flow cytometry analysis outcomes reinforces the potential influence of *R. intestinalis* on macrophage polarization toward an M1 phenotype. This phenomenon is not as pronounced with *P. stomatis* or mix treatment. It is noteworthy that the similarity observed between the M2 control group and the M0 control group in the PCoA plot may be attributed to the colony-stimulating factors used for the differentiation of BMDMs. BMDMs cultivated with macrophage colony-stimulating factor (M-CSF) resulted in a more M2-like phenotype; in contrast, BMDMs cultivated with granulocyte–macrophage colony-stimulating factor (GM-CSF) tended to exhibit an M1-like phenotype [55].

Extensive research has implicated *R. intestinalis* and its derivatives in immune modulation and inflammatory regulation. *R. intestinalis* primarily functions as a butyrate-producing bacterium [31]. Several studies have postulated that the beneficial effects of *R. intestinalis* are mediated through the metabolite butyrate. For example, Ji et al. demonstrated that butyrate facilitates M2 macrophage polarization both in vitro and in vivo. Notably, while butyrate did not directly induce M0 to M2 polarization, it augmented the extent of polarization in IL-4-induced M2 macrophages [56]. Furthermore, *Gao* et al. could show that tumor cell-derived lactate stimulated the M2 polarization of THP-1 cells, which, in turn, further promoted carcinogenicity. However, the inhibition of lactate using 3-hydroxy-butyrate reversed the effects [57]. This stands in contrast to our findings, suggesting that our observed effects may not be dependent on metabolites but most likely result from bacterial components, such as MAMPs.

In co-culture experiments involving MC-38 cells, M0 and bacteria, a significant increase in M1 macrophage differentiation was observed following *R. intestinalis* stimulation. The increase surpassed the effects observed after *P. stomatis* stimulation and the control groups, mirroring the pattern observed in the M0 group without MC-38 cells. In supernatant experiments with M0 macrophages (SN + M0), co-culturing the supernatant of bacteria-stimulated MC-38 cells with M0 macrophages resulted in increased M1 differentiation and a significant decrease in M2 macrophages within the *R. intestinalis* group. Similar effects were observed after stimulation with the mix treatment, leading to increased M1 and decreased M2 macrophages. An increase in M1 macrophages was noted in the *P. stomatis* group, although the specific attribution to the bacterial species remains unclear. These experiments suggest a predisposition towards M1 polarization with *R. intestinalis* treatment in a cancer setting but a more generalized bacteria-unspecific priming effect within MC-38 supernatant experiments. However, it is important to acknowledge that the viability of MC-38 cells remained intact in all conditions, suggesting that anti-cancer processes had not been initiated. This may be attributed to the relatively short experimental duration, as macrophage-mediated tumor cytotoxicity is a gradual, contact-dependent process that typically requires 1–3 days [48]. Furthermore, variations in susceptibility to macrophage-mediated tumor cytotoxicity among different tumor cell lines may be another explanation for the lack of an observed anti-tumor effect. Microsatellite instability–high tumor cell lines might possibly elicit a more rapid and potent macrophage cytotoxic response. Variable results in the co-culture MC-38 supernatant with macrophages experiments may be attributed to a short incubation period, interruptions in chemokine supply within the supernatant experiment setup or suboptimal medium conditions for MC-38 cells.

It is plausible that macrophages are activated by *R. intestinalis* and subsequently exhibit cytotoxicity towards cancer cells when exposed to an additional stimulus from cancer antigens. Macrophages can be activated to exert tumor cytotoxicity through metabolites and MAMPs [58] with LPS, a potent activator from the outer membrane of Gram-negative bacteria, serving as one such trigger for macrophages and monocytes. Notably, the cell wall of Gram-positive bacteria (such as *R. intestinalis*) lacks LPS but instead contains peptidoglycan, which, in turn, shares some properties with LPS, such as glycosylated bacterial microbe-associated molecular patterns [59,60]. Of note, bacterial-derived flagellin, which is also present in *R. intestinalis*, has been demonstrated to induce the activation of pro-inflammatory macrophages, as observed in our own data [61,62]. In our previous in vivo study, the oral administration of *R. intestinalis* alone or in combination with other *Clostridiales* bacteria resulted in enhanced tumor cell killing and reduced levels of PD-L1-positive macrophages in vivo [16]. Another category of macrophage-activating agents includes cytokines. Our experiments indicated that treatment with *R. intestinalis* elicited a distinct pattern of chemokine and cytokine secretion in MC-38 cells, which differed from control conditions and treatment with *P. stomatis*. Specifically, the expression levels of two chemokines, GM-CSF and monocyte chemotactic protein-1 (MCP-1/CCL2), were elevated. GM-CSF, a macrophage-activating cytokine, induces the proliferation of granulocyte and monocyte precursor cells. Additionally, GM-CSF enhances the expression of adhesion molecules, respiratory burst and phagocytic capacity. MCP-1, also known as monocyte chemotactic and activating factor, has been identified as a critical player in recruiting circulating monocytes and attracting macrophages to tumor sites [59,63]. Conversely, no chemokine and cytokine releases were noticed after specific bacterial treatments, neither in M0 cells nor in co-cultures of MC-38 and M0 cells. This might be due to the inability of M0 macrophages to recognize *R. intestinalis* as such. M0 macrophages require prior activation in order to differentiate to M1 after exposure to *R. intestinalis*. This averts autoimmune or pro-inflammatory responses towards the bacterium itself.

*R. intestinalis* appears to induce an immune-activating chemokine cocktail in cancer/epithelial cells, thereby attracting numerous immune cells. It is imperative to evaluate whether this cocktail imparts a pro-inflammatory or anti-inflammatory environment. One plausible mechanism involves epithelial cells, which are known to secrete cytokines to regulate the immune response to infection, inflammation and homeostasis [64]. These cells sense *R. intestinalis* and release chemoattractants for monocytes/macrophages and other immune cells, thereby establishing a robust cancer immune response. The flagellum of *R. intestinalis* facilitates penetration into the colonic mucus layer, enabling interaction with epithelial cells and the subsequent release of chemokines/cytokines. This theory is supported by another research group, which showed that the flagella of *R. intestinalis* stimulate intestinal epithelial cells through TLR5 with subsequent chemokine release [65]. When monocytes/macrophages are recruited, they differentiate or reprogram into the M1 phenotype through *R. intestinalis* stimulation and subsequently target cancer cells. In conclusion, both bacterial MAMPs and specific cytokines can activate monocytes and macrophages to exhibit tumor cytotoxicity [59]. Which of the two mechanisms applies, or whether even both apply, needs to be further investigated.

As demonstrated in our previous research, *R. intestinalis* supplemented via oral gavage effectively treated CRC in mice, enhancing the infiltration and activation of CD8 + T-cells within tumors [16]. This prompted an exploration of the effects of *R. intestinalis* on T-cells in vitro. Although dendritic cells are typically regarded as the primary cell type responsible for generating cytotoxic T-cells, macrophages also possess the capability to stimulate cytotoxic T-cell generation through efficient antigen presentation. Hence, macrophages offer a non-specific, direct mode of tumor cell elimination, as well as a more specific, indirect mechanism [59]. A study by Toujas et al. directly compared macrophages with dendritic cells in generating cytotoxic T-cell clones targeting melanoma and found both cell types to be comparably effective, particularly when loaded with melanoma-specific peptides [66].

However, our investigation into the influence of *R. intestinalis* and *P. stomatis* with macrophages on T-cell response via flow cytometry analysis did not reveal any discernible bacteria-specific effects on T-cell activation or exhaustion, or cytotoxic markers (Author’s unpublished data [39]). Several factors, such as an artificial experimental setup, may have contributed to the absence of observable effects. Shen et al., using a similar setup but with an in vitro model of Crohn’s disease, demonstrated that *R. intestinalis* stimulated thymic stromal lymphopoietin production in intestinal epithelial cells/CaCo-2 through TLR5, ultimately leading to dendritic cell activation and further to T-cell differentiation [65]. Thus, the potential influence of *R. intestinalis* on T-cells may be context-dependent or may require specific conditions and other cell types to manifest.

Another consideration is that macrophages may not have a direct impact on T-cells due to spatial limitations. While dendritic cells are known to migrate to draining lymph nodes to prime T-cells effectively, macrophages are tissue-resident. Given the role of GM-CSF in stimulating macrophages and dendritic cells to enhance antigen presentation to T-cells, its secretion may also play a role in initiating cytotoxic T-lymphocyte induction. Most studies involving tumor cells transfected with GM-CSF have reported an increase in the number of antigen-presenting cells, macrophages and/or dendritic cells and CD4+ and CD8+ T-cells within the tumor [59,67,68,69].

In patients with CRC, the gut experiences a reduction in total bacterial diversity and abundance, resulting in an enrichment of specific bacterial species that enhance macrophage-driven tumorigenic activities. For instance, *F. nucleatum* has been shown to facilitate the recruitment of M2 macrophages and myeloid-derived suppressor cells (MDSCs), contributing to an immunosuppressive TME favoring tumor development and progression [48]. Notably, Kang et al. demonstrated that the administration of *R. intestinalis* significantly inhibited tumor formation, suppressed tumor growth, promoted the presence of cytotoxic CD8+ T-cells and reduced MDSCs. These findings align with our observations and hold promise for *R. intestinalis* as a potential candidate in anti-tumor therapy, especially in the context of macrophage priming and CRC. Nonetheless, further investigations are warranted to comprehensively explain these mechanisms.

Our in vitro approach certainly has its limitations, particularly since it does not reflect the complex TME found in vivo, but also in terms of the limitations of co-culturing cells and anaerobic bacteria (e.g., viability). Nevertheless, when assessing both the limitations of culturing mouse cells under anaerobic conditions or of culturing anaerobic bacteria under aerobic conditions, we finally chose to perform co-cultures under aerobic conditions, even though this might somehow compromise bacterial viability; this caveat must be taken into consideration when interpreting our results regarding immune cell activation. Further, we used murine cells instead of human cells for our study. We acknowledge that the use of human cells might be even more relevant; however, establishing co-culture conditions for primary human cells or organoids might be even more challenging. However, such studies should be conducted in the future to further elucidate the impact of *R. intestinalis* in the context of the human immune response. Nevertheless, we identified an important role for *R. intestinalis* in mediating M1 macrophage polarization. In this regard, our data are well in line with our previously published in vivo data showing that the oral administration of *R. intestinalis* is a highly efficient anti-tumor therapy in a broad number of preclinical cancer models [16]. Thus, our study well supports these experimental tumor therapy approaches and supports the assumption that *R. intestinalis* might indeed be beneficial as an anti-tumor therapy. Thus, future research has to further define this aspect and unravel the molecular mode of action of *R. intestinalis* in mediating anti-tumor immunity, particularly with a focus on T-cells.

## 4. Materials and Methods

### 4.1. Bacterial Culture and Treatment

*Roseburia intestinalis* DSM 14,610 and *Peptostreptococcus stomatis* DSM 17,678 were commercially purchased from DSMZ, Germany. The strains were cultivated at 37 °C in either yeast extract, casitone and fatty acid (YCFA) medium (*R. intestinalis*); or peptone, yeast extract and glucose (PYG) medium (*P. stomatis*) (Supplementary Table S1). Strains were maintained under an anoxic atmosphere using the Hungate technique [70]. The viability of cells was confirmed by flow cytometry using the LIVE/DEAD BacLight bacterial viability kit (Thermo Fisher Scientific Reinach BL, Switzerland) or by Bactobox (SBT Instruments A/S, Herlev, Denmark). For experiments, the optical density of 8 h- to 24 h-old bacterial cultures was measured at 600 nm (OD600) with a cell density meter (Biochrom WPA CO8000, Cambridge, UK). The ratio of the number of bacterial cells to the number of mouse cells was calculated for each bacterial strain, and a multiplicity of infection (MOI) of 10 was used. For the control groups, fresh YCFA medium was used, equivalent to the treatment volume of the *R. intestinalis*-supplemented groups.

### 4.2. Generation and Stimulation of Bone Marrow-Derived Macrophages

Bone marrow-derived macrophages (BMDMs) were generated through the in vitro cultivation of monocytes from murine bone marrow. The collection of murine bone marrow cells, as well as animal husbandry, was performed according to Swiss animal welfare legislation and approved by the local veterinary office (Veterinary Office of the Canton Zürich, Switzerland, approval no: ZH145-2020). The genetic background of all mice used was WT C57BL/6 JRj; they were purchased from Janvier Labs (Le Genest-Saint-Isle, FR, France). Male and female animals 8–12 weeks old were used for all cell isolations. Animals were housed under specific pathogen-free (SPF) conditions with food and water ad libitum. Mice were euthanized with CO_2_, and bone marrow cells were isolated following a previously described method [71]. In brief, bone marrow cells were gently flushed from the femur and tibia bones. These isolated cells were then cultured in complete RPMI 1640 medium (Thermo Fisher Scientific) supplemented with 10% FBS (fetal bovine serum, BioWest S.A.S., Nuaillé, FR, France), 1% sodium pyruvate (Thermo Fisher Scientific), 0.5% penicillin–streptomycin (Thermo Fisher Scientific), 0.5% L-glutamin (Thermo Fisher Scientific) and 5–10% macrophage colony-stimulating factor (M-CSF, harvested from L929 supernatant) for 6 days at a density of 1 × 10^6^ cells/mL in 6- or 12-well non-treated cell culture plates at 37 °C with 5% CO_2_. The medium was replenished on day 3 and renewed on day 5 of the culture period. On day 6, the macrophages were either kept in an undifferentiated M0 state by replacing the culture medium with RPMI 1640 or subjected to M1 differentiation by adding lipopolysaccharide (LPS) (Labforce) at a concentration of 100 ng/mL, alongside murine recombinant interferon-gamma (IFN-γ) (PreproTech, Cranbury, NJ, USA) at 20 ng/mL. For macrophage M2 differentiation, murine recombinant interleukin-4 (IL-4) (PreproTech) was supplemented at 50 ng/mL, along with murine recombinant interleukin-10 (IL-10) (PreproTech) at 40 ng/mL. These M1 and M2 differentiation-inducing factors were incubated with the cells for 24 h. On day 7, the culture medium was again substituted with RPMI 1640, rendering the cells primed and prepared for subsequent bacterial stimulation.

### 4.3. Co-Culture of BMDMs and/or MC-38 Cells with Bacteria

For BMDM co-culture experiments, bacterial stimulation was performed in 6- or 12-well plates containing 1 × 10^6^ and 5 × 10^5^ macrophages in differentiation states M0, M1 or M2, respectively, in RPMI 1640 medium for a 24 h period at 37 °C with 5% CO_2_.

MC-38 cells were cultured at 37 °C with 5% CO_2_ in DMEM high-glucose cell culture medium (Sigma-Aldrich Chemie GmbH, Buchs, SG, Switzerland) containing 10% FBS (BioWest), 1% NEAA (non-essential amino acid solution, Thermo Fisher Scientific) and 1% sodium pyruvate (Thermo Fisher Scientific). For experiments involving bacterial stimulation, MC-38 cells were seeded in 6- or 12-well plates at a density of 2 × 10^5^ and 1 × 10^5^ cells per well, respectively. After 24 h, the culture medium was replaced with RPMI 1640, and bacterial stimulation was applied for an additional 24 h. Following the bacterial stimulation of MC-38 cells, the supernatant was collected and subsequently centrifuged at 10,000 rpm for 10 min at 4 °C. The supernatant was either used for multiplex analysis or to stimulate BMDMs for 24 h.

For BMDMs and MC-38 cell co-culture experiments, 1 × 10^5^ and 5 × 10^4^ MC-38 cells were added to 6- or 12-well plates containing 1 × 10^6^ and 5 × 10^5^ M0 differentiated macrophages, respectively, in RPMI 1640 medium. Subsequently, these co-cultures were directly exposed to bacterial treatment for a 24 h period and utilized for subsequent analytical procedures.

### 4.4. Flow Cytometry

Cell culture experiments first involved a cell-washing step with PBS and a subsequent restimulation for a period of 3 h at 37 °C in RPMI 1640 containing 10% FBS, PMA (50 ng/mL, Sigma-Aldrich), Ionomycin (1 µg/mL, Sigma-Aldrich) and Brefeldin A (1 µg/mL, Sigma-Aldrich). A cell-washing step with PBS and detachment of the cells using accutase (Innovative Cell Technology, Ebnat-Kappel, Switzerland) for 5–20 min at 4 °C was followed by further collection and centrifugation at 1500 rpm for 5 min at 4 °C.

The cell suspensions were incubated with primary antibodies in PBS for a duration of 20 min at 4 °C, followed by a PBS washing step and centrifugation at 1500 rpm for 5 min at 4 °C. To fix and permeabilize the cells, BD Cytofix/Cytoperm^TM^ (BD Biosciences, Eysins, VD, Switzerland) was used for 20 min at 4 °C, then the cells were washed with Perm buffer (BD Biosciences) and centrifuged at 1500 rpm for 5 min at 4 °C. Subsequent intracellular staining was carried out in Perm buffer for 20 min at 4 °C, followed by another washing step with Perm buffer and a centrifugation step. For intranuclear staining, cells were fixed and permeabilized with Transcription Factor Staining Buffer (eBioscience, Zug, Switzerland), followed by a wash with Perm Buffer (eBioscience) and a centrifugation step before staining with intranuclear antibodies in Perm Buffer. Staining was completed with a final PBS washing step.

For data acquisition, samples were resuspended in PBS and analyzed by flow cytometry on a BD FACSymphony equipped with 355 nm, 405 nm, 488 nm, 561 nm and 639 nm laser lines with FACS Diva Software (v9.0, BD Biosciences, Franklin Lakes, NJ, USA). Prior to data acquisition, photomultiplier tube (PMT) voltages were adjusted manually to minimize fluorescence spillover, and single-stain controls were acquired for compensation matrix calculation. A list of the antibodies used is given in Supplementary Table S2. Samples were analyzed using the FlowJo software (v9.0, BD Biosciences, Ashland, OR, USA), employing a standardized gating approach. Cells were initially identified using forward scatter (FSC) and side scatter (SSC) gates. Singlets were selected using the FSC-H and FSC-A gate. Live immune cells were subsequently chosen based on the CD45+ Zombie NIR-gate.

Macrophage panel: macrophages were gated as F4/80+ CD64+ cells. Macrophages were further subtyped into M1 as CD80+/− CD86 + and M2 as CD206+ CD163+/−. Additionally, PDL-1+, PDL-2+, IFNγ+ or TNFα+ cells were analyzed within the M1 and M2 populations.

### 4.5. RNA Extraction and RT-PCR

Cells were harvested and snap frozen. RNA was isolated using the Maxwell RSC simplyRNA Tissue Kit (Promega, Madison, WI, USA) according to the manufacturer’s instructions. The RNA concentration was determined using a Biotek synergy H1 plate reader through the measurement of absorbance at 260 nm. Complementary DNA (cDNA) was synthesized using the high-capacity cDNA Reverse Transcription Kit (Thermo Fisher Scientific), following the manufacturer’s instructions. Quantitative PCR (qPCR) was performed using FAST qPCR Master Mix and pre-designed TaqMan assays (*Arg1*, Mm00475988_m1; *Nos2*, Mm00440502_m1; *Cd80*, Mm00711660_m1; *Cd86*, Mm00444543_m1; *Mrc1* (*Cd206*), Mm01329362_m1; *Cd163*, Mm00474091_m1; *IL-1b*, Mm01336189_m1, Thermo Fisher Scientific). Mouse *Gapdh* (4352339E, Thermo Fisher Scientific) was used as the endogenous control, measurements were performed in triplicate and relative expression levels were calculated according to the ΔΔCt method.

### 4.6. Bio-Plex Multiplex Immunoassay

The levels of chemokines and cytokines in the supernatant obtained from MC-38 cells stimulated with bacteria, M0 cells stimulated with bacteria or co-cultures of MC-38 and M0 cells stimulated with bacteria were analyzed. The supernatant was collected, snap frozen and stored at -20 C until processed. The high-throughput multiplex assay Bio-Plex Pro Mouse Chemokine Panel 31-Plex (12009159, BioRad, Cressier, FR, Switzerland) was used to measure the chemokine and cytokine levels of BCA-1/CXCL13, CTACK/CCL27, ENA-78/CXCL5, Eotaxin2/CCL24, Fractalkine/CX3CL1, GM-CSF, I-309/CCL1, IP-10/CXCL10, I-TAC/CXCL11, IL-1β, IL-2, IL-4, IL-6, IL-10, IL-16, KC/CXCL1, MCP-1/CCL2, MCP-3/CCL7, MCP-5/CCL12, MDC/CCL22, MIP-1a/CCL3, MIP-1b/CCL4, MIP-3a/CCL20, MIP-3b/CCL19, RANTES/CCL5, SCYB16/CXCL16, SDF-1a/CXCL12, TARC/CCL17, IFN-γ and TNF-α. Samples were processed following the manufacturer’s instructions and measured with the Bio-Plex 200 system (BioRad).

### 4.7. RNA Sequencing and Data Analysis

For RNA sequencing analysis, BMDMs with an M0, M1 or M2 phenotype and YCFA medium as the control, *R. intestinalis*, *P. stomatis* or a mix stimulation of *R. intestinalis* and *P. stomatis* were used. RNA was isolated as described previously, and quality was assessed using the Agilent 2100 Bioanalyzer (Agilent Technologies, Inc., Basel, CH, Switzerland). RNA samples with an RNA integrity number (RIN) above 8.5 were used for library preparation. Libraries were prepared using the TruSeq Stranded mRNA Sample Preparation Kit (Illumina, Inc., Basel, CH, Switzerland) following the manufacturer’s instructions. Sequencing was performed by the *Functional Genomics Center Zurich* (FGCZ) using Illumina instruments (Illumina Switzerland, Basel, Switzerland). The raw sequencing data were first subjected to quality control using fastp version 0.23.4 to trim adapters and filter out low-quality reads. Mouse genome mm10 from Ensemble was used as a reference genome for mapping the reads with STAR (version 2.7.3a). To quantify gene expression levels, the Subread package version 2.0.3 was applied. The DESeq2 R package (version 1.28.1) was utilized for detecting differentially expressed genes. The negative binomial model and Wald test were performed with the Benjamini–Hochberg for multiple comparisons correction (FDR cut-off < 0.05). The Database for Annotation, Visualization, and Integrated Discovery (DAVID) was utilized for functional annotation, and the Goplot package version 1.0.2 was used for visualization.

### 4.8. Statistical Analysis

All statistical analysis and visualization, except for the RNA sequencing data, were performed using GraphPad Prism v.9 (GraphPad Software). A non-parametric two-tailed Mann–Whitney test was used to compare the two groups. A non-parametric Kruskal–Wallis test was used to compare three or more groups and apply TukeyHSD post hoc tests. The significance level was set at * *p* < 0.05, ** *p* < 0.01, *** *p* < 0.001 and **** *p* < 0.0001.

## 5. Conclusions

From a broader perspective, our understanding of *R. intestinalis* remains limited, primarily due to a lack of comprehensive studies beyond the mere detection of its presence. It is possible that the metabolites produced by *R. intestinalis* exert a more potent or synergistic influence, warranting further investigation. *R. intestinalis* could potentially influence immune cell functions and cytokine release through the action of its metabolites, such as butyrate, as well as other yet unidentified components of the supernatant. We anticipate that advancements in the techniques of primary culture, culturomics, single-cell sequencing, and metabolomics will play pivotal roles in unraveling the intricate role of *R. intestinalis*. This, in turn, will establish a robust foundation for understanding *R. intestinalis*’s significance in maintaining human health and developing effective strategies for disease treatment in the future.

## Figures and Tables

**Table 1:** Bio-Plex multiplex immunoassay of macrophages and MC-38 cells. Chemokine and cytokine expression levels of supernatant from MC-38 cells, M0 or MC-38 cells + M0 co-cultured with *R. intestinalis* or *P. stomatis* measured with a Bio-Plex multiplex immunoassay. The (>) symbol indicates an increase (not significant) compared to the control group. Two different experiments were performed, consisting of *n* = 3 in all groups. The data presented are from one representative subset. *p*-value was determined with Kruskal–Wallis and Dunn’s correction for multiple comparison with * *p* < 0.05, ** *p* < 0.01, *** *p* < 0.001 and **** *p* < 0.0001.

	MC-38		M0		MC-38 + M0	
	*R. intestinalis*	*P. stomatis*	*R. intestinalis*	*P. stomatis*	*R. intestinalis*	*P. stomatis*
BCA-1/CXCL13	>		>	>	>	>
CTACK/CCL27	> *		> *	> *	>	>
ENA-78/CXCL5	>		> **	> **	> ***	> ***
Eotaxin2/CCL24					>	>
Fractalkine/CX3CL	>		> **	> **	> **	> **
GM-CSF	> **					
I-309/CCL1	>		>	>	>	>
I-TAC/CXCL11	>		> *	> **	>	> *
IFN-γ						
IL-1β	>		> *	>	> *	> **
IL-2						
IL-4	>		> **	> **	>	>
IL-6			>	>		
IL-10			> **	> **	> **	>
IL-16			>	> *		
IP-10/CXCL10	>		>	>	> *	>
KC/CXCL1	> **		>	> *	>	>
MCP-1/CCL2	> **		> **	> *	> **	> *
MCP-3/CCL7	>		> **	> ***	> *	> *
MCP-5/CCL12			>	> **	> **	> ****
MDC/CCL22	>		> **	> *	>	> ***
MIP-1a/CCL3	>		>	>	>	>
MIP-1b/CCL4	> **		>	>		>
MIP-3a/CCL20	> **		> **	> **	>	> *
MIP-3b/CCL19			> *	> **	> *	> ***
RANTES/CCL5			>	>	>	>
SCYB16/CXCL16					>	>
SDF-1a/CXCL12			> ***	> ***	> **	> ****
TARC/CCL17			> ***	> ***	> **	> ***
TNF-α	> **		> *	> ****		> **

## Data Availability

The raw sequencing data presented in this study are available from the NCBI bioproject via the accession PRJNA1090008. https://www.ncbi.nlm.nih.gov/bioproject/ (accessed on 12 May 2025). Data have been deposited at NCBI Sequence Read Archive (SRA) and are publicly available as of the date of publication. The accession number for the database is PRJNA1090008. Any additional information required to reanalyze the data reported in this paper is available from the lead contact upon request.

## Supplementary Materials

The following supporting information can be downloaded at: https://www.mdpi.com/article/10.3390/ijms26115049/s1.

## Abbreviations

ACK	Ammonium–chloride–potassium
ARG1	Arginase 1
BMDMs	Bone marrow-derived recruited macrophages
CRC	Colorectal cancer
CTLA-4	Cytotoxic T-lymphocyte-associated protein 4
DEG	Differentially expressed genes
FBS	Fetal bovine serum
FGCZ	Functional Genomics Center Zurich
GI	Gastrointestinal
GM-CSF	Granulocyte–macrophage colony-stimulating factor
GO	Gene Ontology
IFN-γ	Interferon-gamma
IL	Interleukin
iNOS	Inducible nitric oxide synthase
LPS	Lipopolysaccharide
MAMPs	Microbial-associated molecular patterns
MCP-1	Monocyte chemotactic protein-1
M-CSF	Macrophage colony-stimulating factor
MDSC	Myeloid-derived suppressor cells
MOI	Multiplicity of infection
NEAA	Non-essential amino acid
NOS2	Nitric oxide synthase 2
OD	Optical density
PD-1	Programmed cell death protein 1
PD-L1	Programmed cell death protein ligand 1
PRRs	Pattern recognition receptors
PYG	Peptone, yeast extract and glucose
RNI	Reactive nitrogen intermediates
ROS	Reactive oxygen species
TAMs	Tumor-associated macrophages
TLR	Toll-like receptor
TME	Tumor microenvironment
TNFα	Tumor necrosis factor alpha
YCFA	Yeast extract, casitone and fatty acid
