# Supplementary material for: Roseburia intestinalis Modulates Immune Responses by Inducing M1 Macrophage Polarization
Source: Int J Mol Sci. 2025 May 23;26(11):5049. doi: 10.3390/ijms26115049 (PMC12155563; doi:10.3390/ijms26115049)
Supplement: Supplementary file 1 [file ijms-26-05049-s001.zip › Figure S1.pdf]

A. M0

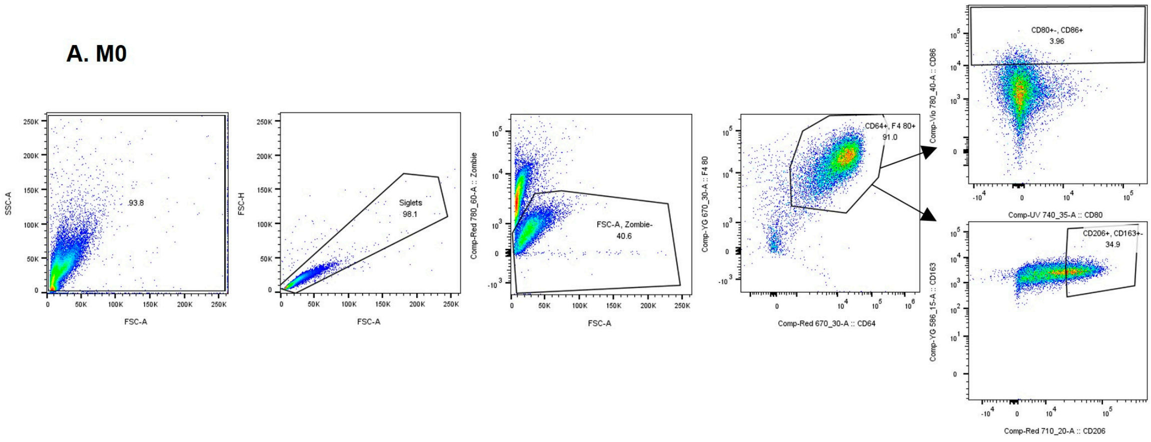

B. M1

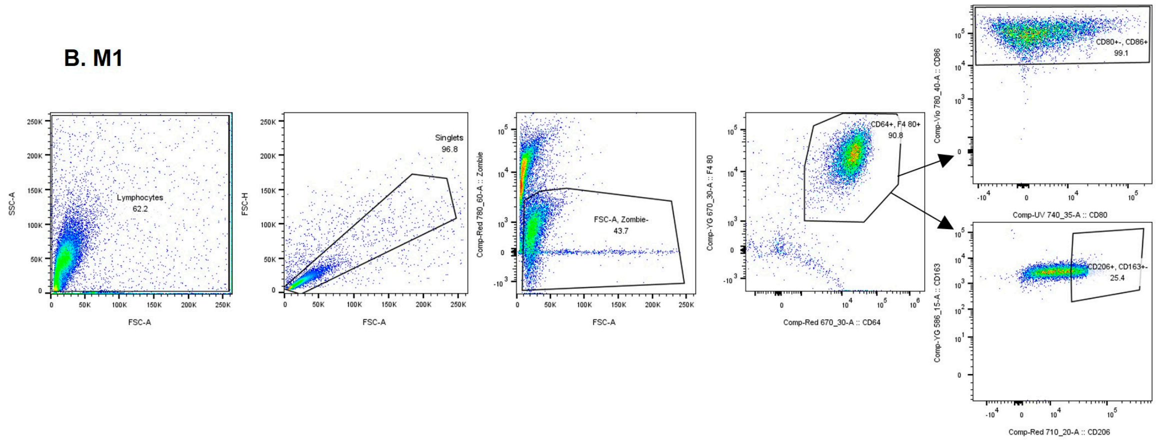

C. M2

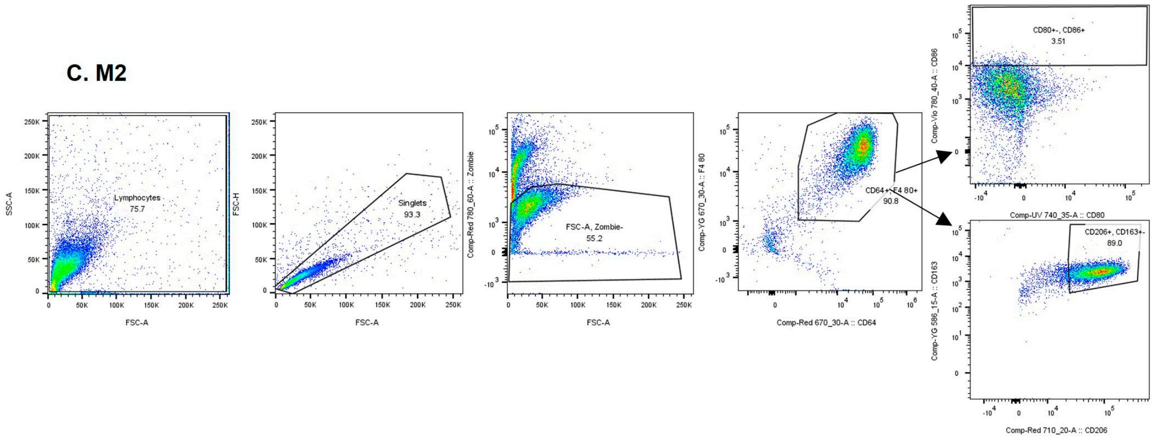

D. MC-38 + M0

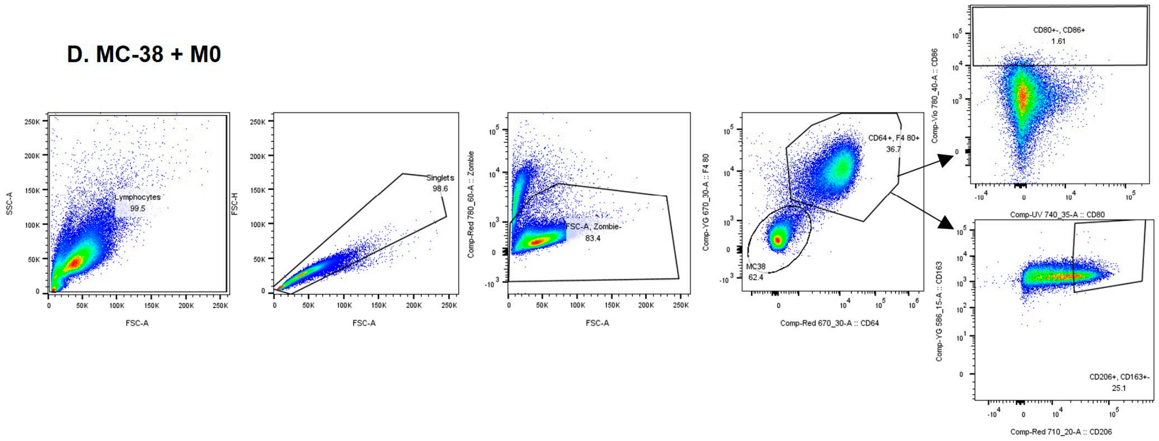

Figure S1: Gating strategy for flow cytometry. Example analysis of cell culture experiments with **A.)** M0 **B.)** M1, **C.)** M2 macrophages and **D.)** co-cultures of MC-38 and M0 macrophages stimulated with *R. intestinalis, P. stomatis* or a mix of both, compared to control group.
