# Supplementary material for: Roseburia intestinalis Modulates Immune Responses by Inducing M1 Macrophage Polarization
Source: Int J Mol Sci. 2025 May 23;26(11):5049. doi: 10.3390/ijms26115049 (PMC12155563; doi:10.3390/ijms26115049)
Supplement: Supplementary file 1 [file ijms-26-05049-s001.zip › Figure S2.pdf]

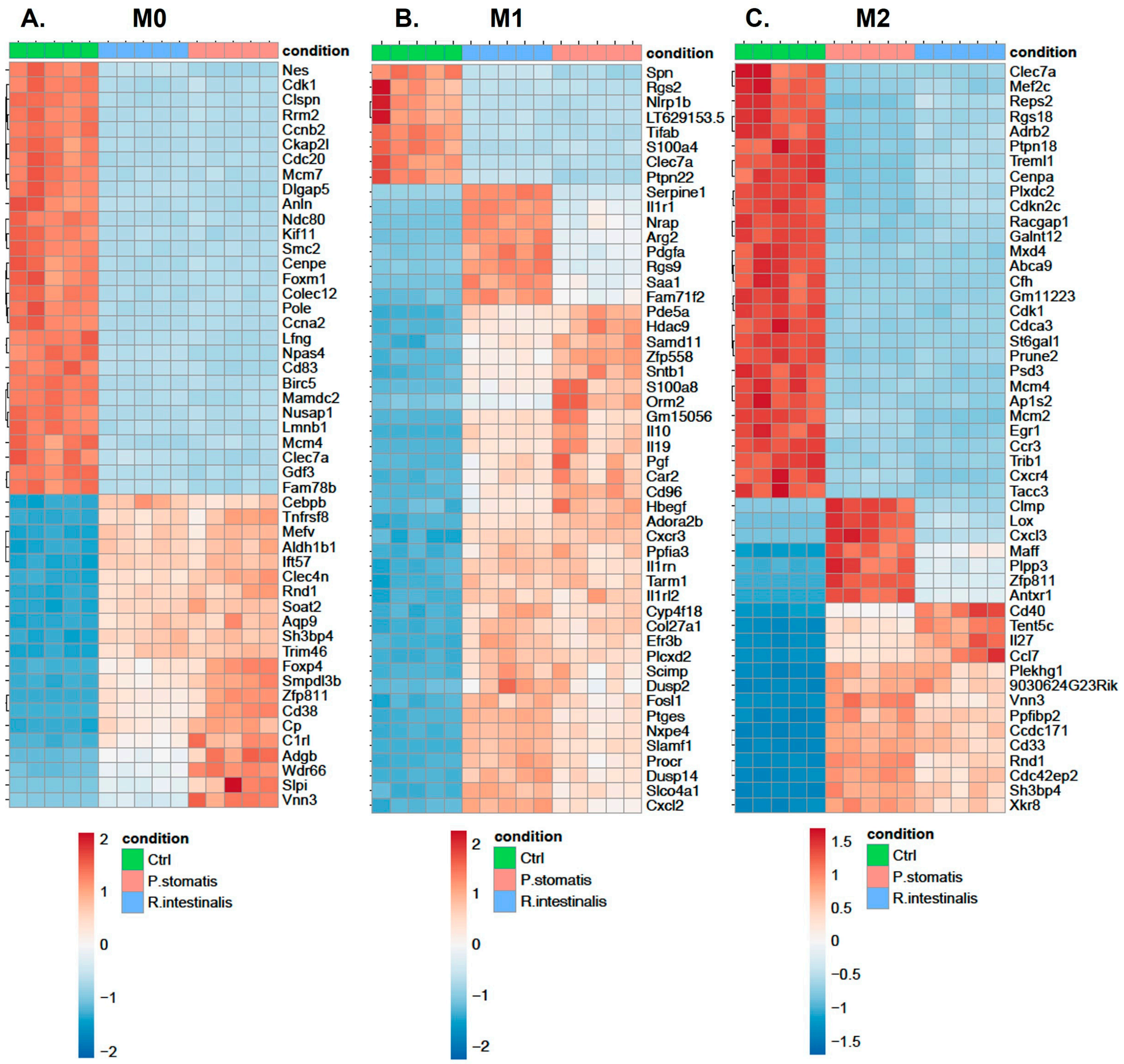

Figure S2: RNA sequencing analysis with Heatmap of the top 50 intersected genes of M0, M1 and M2 differentiated bacterial treated macrophages. Heatmaps showing the top intersected expressed genes of **A.)** M0 **B.)** M1 and **C.)** M2 macrophages stimulated with *R. intestinalis* or *P. stomatis*, compared to control group.
