# Supplementary material for: Roseburia intestinalis Modulates Immune Responses by Inducing M1 Macrophage Polarization
Source: Int J Mol Sci. 2025 May 23;26(11):5049. doi: 10.3390/ijms26115049 (PMC12155563; doi:10.3390/ijms26115049)
Supplement: Supplementary file 1 [file ijms-26-05049-s001.zip › Figure S3.pdf]

MC-38 cells and primary mouse BMDMs are not sufficiently viable under anoxic condition

To enable co-cultures of anaerobic bacteria and MC-38 cells or BMDMs, we first determined the viability of these cells over time under anoxic conditions. The results indicated that the cells remain viable for up to 6 h in an anoxic environment (Supplementary Figure 1). Signs of detachment started to become visible after 8 h, and after 24 h mark, all cells were dead, as indicated by change in morphology, change of pH in media, and floating cells in the culture. Due to the very limited viability of MC-38 cells and BMDMs in an anoxic environment, all subsequent co-culture experiments were executed under aerobic conditions. However, this approach compromised the viability of the anaerobic bacterial culture.

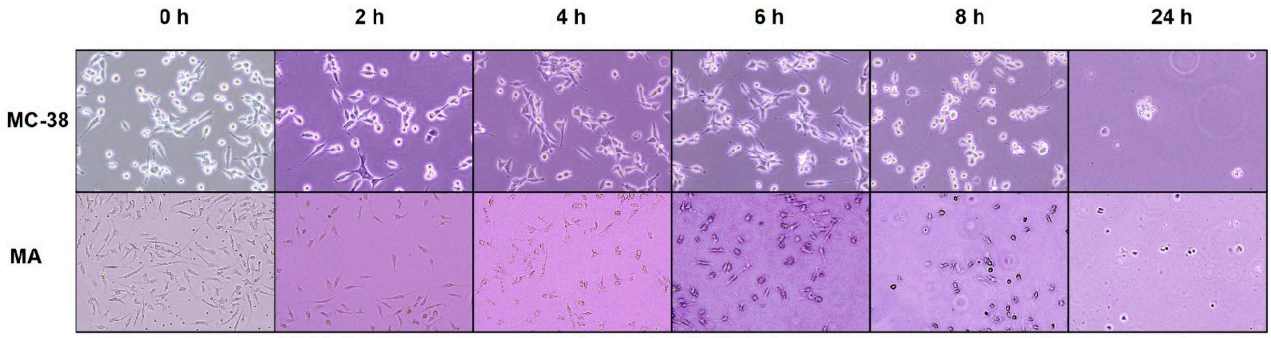

Figure S3: Viability of MC-38 cells and BMDMs from different time points in anoxic environment. Representative pictures of MC-38 cells and BMDMs, under anaerobic condition and 40x magnification.
