# Supplementary material for: Roseburia intestinalis Modulates Immune Responses by Inducing M1 Macrophage Polarization
Source: Int J Mol Sci. 2025 May 23;26(11):5049. doi: 10.3390/ijms26115049 (PMC12155563; doi:10.3390/ijms26115049)
Supplement: Supplementary file 1 [file ijms-26-05049-s001.zip › Table S1.pdf]

Table S1: **List of ingredients for YCFA and PYG bacteria culturing medium.**

Ingredients	YCFA medium medium	PYG medium Medium
Bacto Casitone (Thermo Fisher Scientific)	10g/L	-
Yeast extract (Sigma-Aldrich)	2.5g/L	10g/L
Peptone from casein, tryptic digest (Sigma-Aldrich)	-	5g/L
Meat extract (Sigma-Aldrich)	-	5g/L
Bacto Soytone (Thermo Fisher Scientific)	-	5g/L
L-Cysteine hydrochloride hydrate (Sigma-Aldrich)	1g/L	0.5g/L
Glucose (Sigma-Aldrich)	6g/L	5g/L
Dipotassium phosphate (Roth)	0.45g/L	2.04g/L
Potassium dihydrogen phosphate (Roth)	0.45g/L	40mg/L
Sodium chlorid (Sigma-Aldrich)	0.9g/L	80mg/L
Ammonium sulfate (Sigma-Aldrich)	0.9g/L	-
Magnesium sulfate (Sigma-Aldrich)	90mg/L	-
Calcium chloride (Sigma-Aldrich)	90mg/L	-
Acetic Acid (Sigma-Aldrich)	1.9mL/L	-
Magnesium sulfate heptahydrate (Sigma-Aldrich)	-	20mg/L
Tween 80 (Sigma-Aldrich)	-	1mL/L
Sodium bicarbonate (Sigma-Aldrich)	4g/L	0.4g/L
Calcium chloride dihydrate (Sigma-Aldrich)	-	10mg/L
Resazurin (Sigma-Aldrich)	1mg/L	1mg/L
Hemin (Sigma-Aldrich)	10mg/L	5mg/L
Biotine (Sigma-Aldrich)	0.02mg/L	-
Cobalamin (Sigma-Aldrich)	0.001mg/L	-
p-Aminobenzoic acid (Sigma-Aldrich)	0.05mg/L	-
Folic acid (Sigma-Aldrich)	0.02mg/L	-
Pyridoxine-HCI (Sigma-Aldrich)	0.1mg/L	-
Thiamine-HCI x 2 H_2O (Sigma-Aldrich)	0.5mg/L	-
Vitamine K_1 (Sigma-Aldrich)	-	0.05mL/L
Riboflavin (Sigma-Aldrich)	0.5mg/L	-
Nicotinic acid (Sigma-Aldrich)	0.5mg/L	-
D-Ca-pantothenate (Sigma-Aldrich)	0.5mg/L	-
Lipoic acid (Sigma-Aldrich)	0.5mg/L	-
dd H_2O	+	+
