# Supplementary material for: Roseburia intestinalis Modulates Immune Responses by Inducing M1 Macrophage Polarization
Source: Int J Mol Sci. 2025 May 23;26(11):5049. doi: 10.3390/ijms26115049 (PMC12155563; doi:10.3390/ijms26115049)
Supplement: Supplementary file 1 [file ijms-26-05049-s001.zip › Table S2.pdf]

Table S1: List of used flow cytometry antibodies.

Fluorochrome	Antigen	Clone	Supplier	Category	Order number
PE	CD163	EPR19518	Abcam	intracellular	182422-100
AF700	CD206	MMR	Thermo Fisher Scientific	intracellular	56-2061-82
BUV661	CD3	145-2C11	BD Biosciences	surface/intracellular	750638
BUV563	CD4	GK1.5	BD Biosciences	surface/intracellular	612923
BUV395	CD45	30-F11	BD Biosciences	surface	564279
APC	CD64	X54-5/7.1	BioLegend	surface	139306
BUV805	CD8	53-6.7	BD Biosciences	surface/intracellular	612898
BUV737	CD80	16-10A1	BD Biosciences	surface	612773
BV785	CD86	GL1	BioLegend	surface	105043
BV605	CTLA4 (CD152)	UC10-4B9	BioLegend	surface	106323
PE-Cy5	F4/80	BM8	BioLegend	surface	123112
PE	FoxP3	MF-14	BD Biosciences	intranuclear	72-5775-40
PerCP-Cy5.5	Granzyme B	QA16A02	BioLegend	intranuclear	372212
APC	IFNγ	XMG1.2	BioLegend	intracellular	505810
BV510	IL17A	TC11-18H10.1	BioLegend	intracellular	506933
BV711	IL4	11B11	BioLegend	intracellular	504133
BV570	Ly6C	HK1.4	BioLegend	surface	128030
BV711	Ly6C	HK1.4	BioLegend	surface	128037
AF700	MHC II	(M5)114.15.2	BioLegend	surface	107622
FITC	MHC II	(M5/114.15.2	Thermo Fisher Scientific	surface	11-5321-82
eFluor 450	PD-1 (CD279)	J43	Thermo Fisher Scientific	surface	48-9985-82
BV480	PDL-1 (CD274)	MIH1	BD Biosciences	surface	746275
PE-Cy7	PDL-1 (CD274)	B7-H1	Thermo Fisher Scientific	surface	25-5982-82
PE/Dazzle 594	PDL-2 /CD273)	TY25	BioLegend	surface	107216
FITC	Perforin	eBioOMAK-D	Thermo Fisher Scientific	intranuclear	11-9392-82
BV785	TNFα	MP6-XT22	BioLegend	intracellular	506341
AP-Cy7	Zombie NIR		BioLegend		423106
